# Hydroarylations by cobalt-catalyzed C–H activation

**DOI:** 10.3762/bjoc.14.202

**Published:** 2018-08-29

**Authors:** Rajagopal Santhoshkumar, Chien-Hong Cheng

**Affiliations:** 1 Department of Chemistry, National Tsing Hua University, Taiwan

**Keywords:** catalysis, C−C formation, C–H activation, cobalt, hydroarylation

## Abstract

As an earth-abundant first-row transition metal, cobalt catalysts offer a broad range of economical methods for organic transformations via C–H activation. One of the transformations is the addition of C–H to C–X multiple bonds to afford alkylation, alkenation, amidation, and cyclization products using low- or high-valent cobalt catalysts. This hydroarylation is an efficient approach to build new C–C bonds in a 100% atom-economical manner. In this review, the recent developments of Co-catalyzed hydroarylation reactions and their mechanistic studies are summarized.

## Introduction

For the last three decades, atom-economical synthetic approaches have played a substantial role in organic synthesis owing to the necessity of green chemistry for the modern universe [1–3]. In this context, catalytic C–H functionalization has been acknowledged as an atom- and step-economical process [4–6]. A wide range of transition metal-catalyzed non-directed or directing group assisted C–H activation methodologies have been developed to build new C–X (X = carbon or heteroatom) bonds [7–10] and they offer efficient routes to the synthesis of natural products, materials, agrochemicals, polymers, and pharmaceuticals [11–15]. Specifically, the first-row transition metal catalysts, which are less expensive and more environmentally benign, have attracted significant attention in recent years [16–27]. As a member of the first-row transition metals, cobalt complexes are known to be extensively involved in homogeneous catalysis, in particular, C–H activation.

**Scheme 1:**
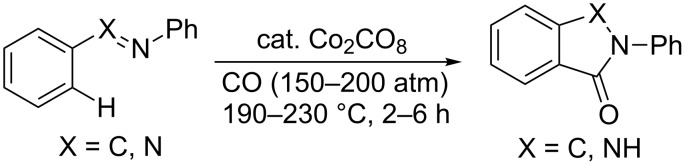
Cobalt-catalyzed C–H carbonylation.

In 1941, Kharasch and Fields applied a cobalt salt as the catalyst for the homocoupling of Grignard reagents [28]. After 15 years, Murahashi discovered a cobalt-catalyzed chelation-assisted *ortho* C–H carbonylation of azobenzene and imines as the preliminary example of directing group assisted C–H activation reactions (Scheme 1) [29–30]. Following to these pioneering works, the groups of Kochi [31], Kisch [32], Klein [33–34], and Brookhart [35] have made their crucial contributions in the cobalt-mediated/catalyzed C–H functionalization. In recent years, Yoshikai [36], Kanai/Matsunaga [37], and Daugulis [38] introduced different cobalt systems, which played a significant portion in C–H activation by its unique reactivity as an alternative to third-row noble metal catalysts [16–17,20–25]. Nakamura [39], Ackermann [40], and Glorius [41] also involved in cobalt-catalyzed C–H functionalization. Of these reactions, alkylation, alkenation, amidation, and cyclization of arenes with the relevant coupling partners are an economical and straightforward approach for the synthesis of diverse alkyls, alkenes, amides and cyclic compounds.

**Scheme 2:**
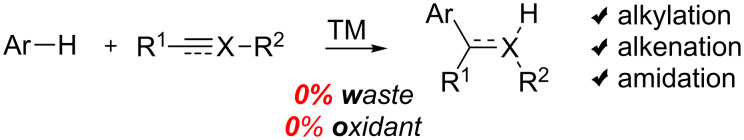
Hydroarylation by C–H activation.

**Scheme 3:**
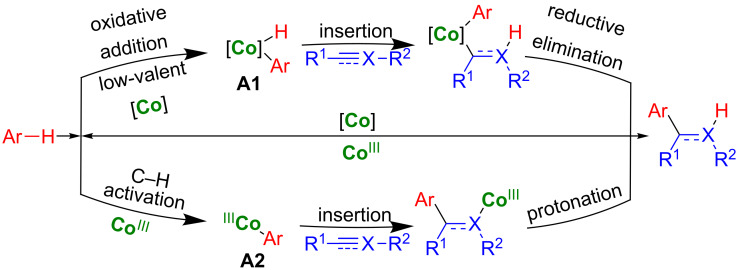
Pathways for cobalt-catalyzed hydroarylations.

A simple addition of a “inert” C–H bond to multiple bonds (hydroarylation) is a 100% atom economical process to build fundamental alkyls and alkenes (Scheme 2) [42–45]. It is an efficient alternative to C–H alkylation reactions with alkyl halides where one equivalent of salt waste was released, and dehydrogenative Heck coupling with alkenes for the synthesis of alkenes, which required a stoichiometric amount of oxidant. Herein, we wish to review the cobalt-catalyzed hydroarylation of alkynes, alkenes, allenes, enynes, imines, and isocyanates. These reactions usually proceed via either an oxidative addition of Ar–H to a low-valent cobalt to form **A1** intermediate or a C–H activation with high-valent cobalt to give **A2** via deprotonation, followed by migratory insertion and reductive elimination or protonation (Scheme 3). We believe that this review will be helpful to the researchers for their future research on hydroarylation using earth-abundant metal catalysts.

## Review

### 1. Hydroarylation of alkynes

#### 1.1 Low-valent cobalt-catalyzed hydroarylation of alkynes

**Scheme 4:**
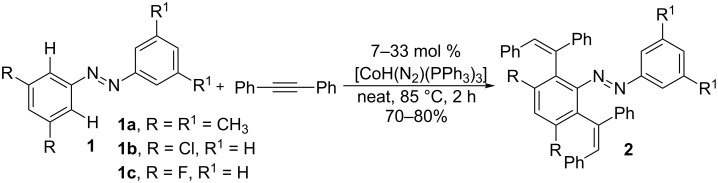
Co-catalyzed hydroarylation of alkynes with azobenzenes.

Hydroarylation of alkynes is an efficient method to synthesize aromatic alkenes in a highly atom-economical way [44]. In 1994, Kisch and co-workers developed the first cobalt-catalyzed hydroarylation of alkynes with azobenzenes **1** to synthesize dialkenated products **2** (Scheme 4) [32]. The reaction resulted in an *anti*-addition of the C–H bond with alkynes using the cobalt(I) catalysts CoH(N_2_)(PPh_3_)_3_ or CoH_3_(PPh_3_)_3_ under neat reaction conditions.

**Scheme 5:**
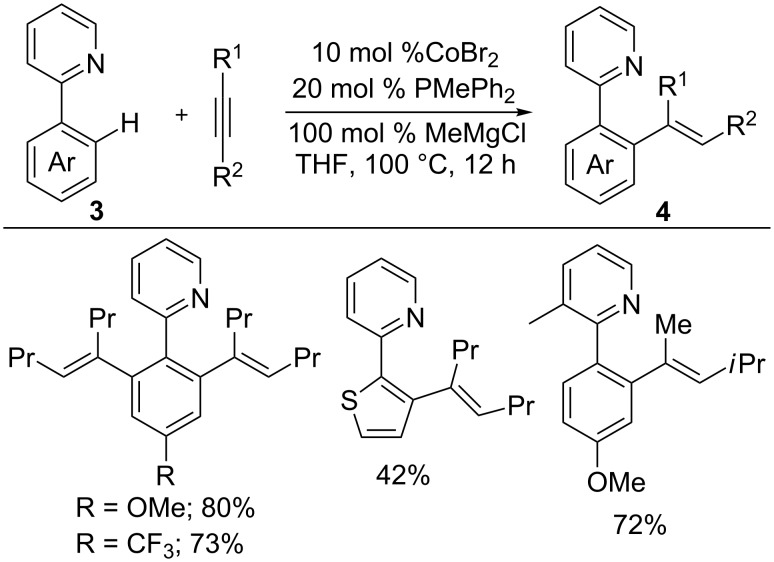
Co-catalyzed hydroarylation of alkynes with 2-arylpyridines.

After fifteen years, Yoshikai and co-workers developed a low-valent cobalt system for C–H functionalization [16,36]. Thus, the reaction of 2-aryl pyridines **3** with internal alkynes in the presence of 10 mol % CoBr_2_, 20 mol % PMePh_2_, and 1 equiv of MeMgCl as a reductant yielded *ortho* alkenated products **4** with high regio- and stereoselectivities (Scheme 5) [36]. The found intermolecular kinetic isotope effect (KIE) of *k*_H_/*k*_D_ = 2.1 and H/D crossover studies strongly suggest that the reaction proceeds through an oxidative addition of a C–H bond to low-valent cobalt followed by alkyne insertion and reductive elimination. Furthermore, the new C–C bond formation occurred at the less-hindered carbon of the unsymmetrical alkynes, which causes the high regioselectivity.

**Scheme 6:**
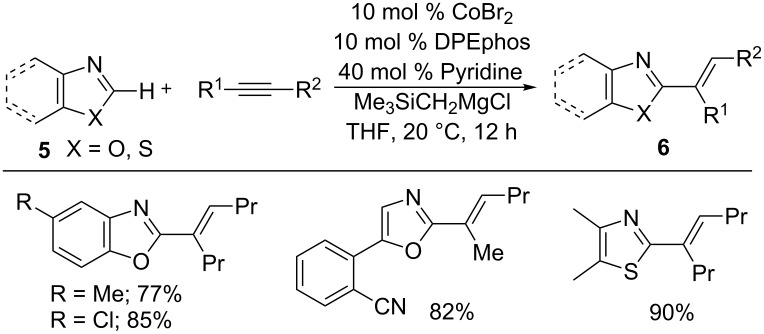
Co-catalyzed addition of azoles to alkynes.

**Scheme 7:**
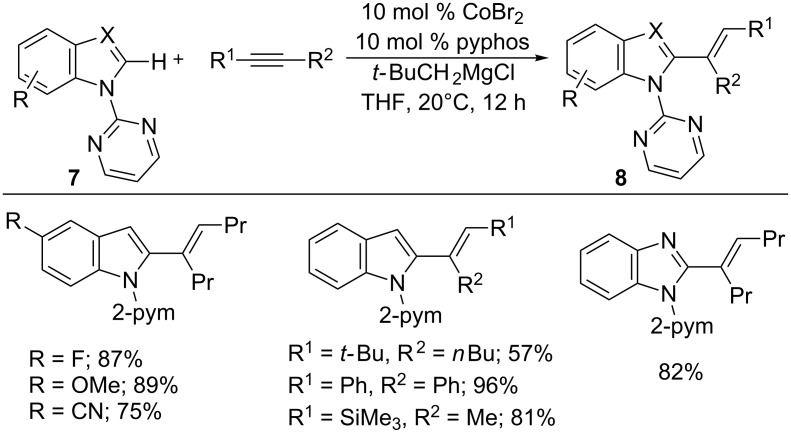
Co-catalyzed addition of indoles to alkynes.

Later, they proved that the hydroarylation reaction was also feasible with benzoxazoles **5** to form alkenated products **6** using CoBr_2_, bis[(2-diphenylphosphino)phenyl] ether (DPEphos), Grignard reagent, and pyridine as an additive (Scheme 6) [46]. Similarly, benzothiazoles also efficiently underwent hydroheteroarylation by using suitable ligands [47]. The reaction was successfully extended to indoles and benzimidazoles **7** bearing a removable 2-pyrimidyl (2-pym) directing group with alkynes at ambient temperature (Scheme 7) [48]. These reactions resemble the Ni(0)-catalyzed hydroheteroarylation reaction [49].

**Scheme 8:**
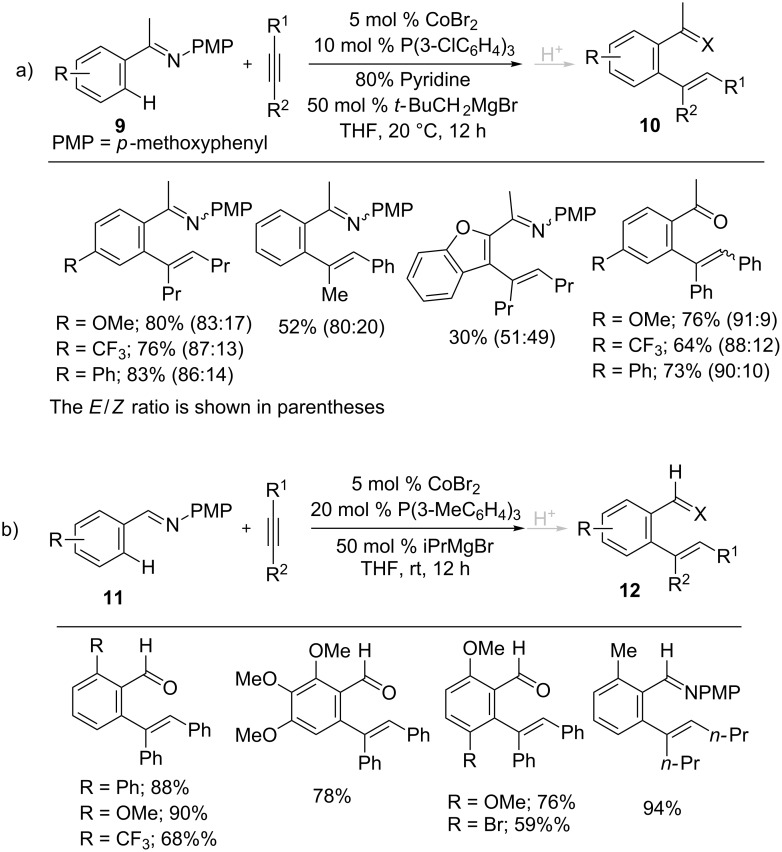
Co-catalyzed hydroarylation of alkynes with imines.

**Scheme 9:**
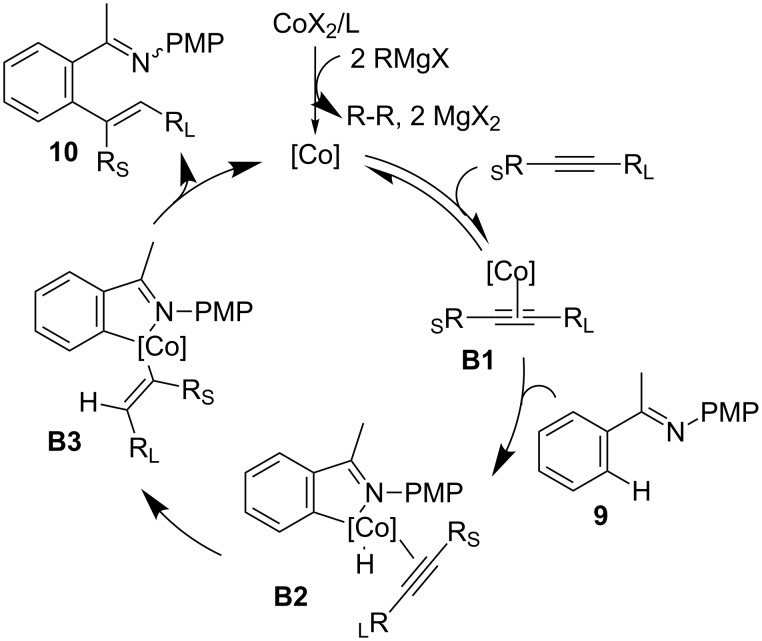
A plausible pathway for Co-catalyzed hydroarylation of alkynes.

In addition, imines **9** and **11** bearing a *p*-methoxyphenyl (PMP) group were also treated with internal alkynes in the presence of a cobalt catalyst generated from CoBr_2_, P(3-ClC_6_H_4_)_3_, and *t-*BuCH_2_MgBr at room temperature to give trisubstituted alkenes **10** and **12** in good yields (Scheme 8) [50–51]. The reaction featured a broad scope of alkynes and imines. The *ortho* alkenated imines could be further transformed into useful products, such as aminoindanols and benzofulvenes under hydrolysis conditions. Based on the mechanistic studies, a possible reaction mechanism for the hydroarylation reaction was proposed in Scheme 9. The reaction begins with the generation of an ambiguous low-valent cobalt catalyst from the reaction of CoBr_2_, ligand and Grignard reagent, which gives the alkane and MgX_2_ as the by-products. Then, coordination of the alkyne with the cobalt catalyst afforded **B1** and the oxidative addition of C–H gave the cobalt complex **B2**. Intramolecular insertion of the Co–H bond into the alkyne and subsequent reductive elimination of the less-hindered alkenyl carbon with aryl group in **B3** provides the desired alkene **10** and regenerates the active cobalt catalyst for the next cycle.

**Scheme 10:**
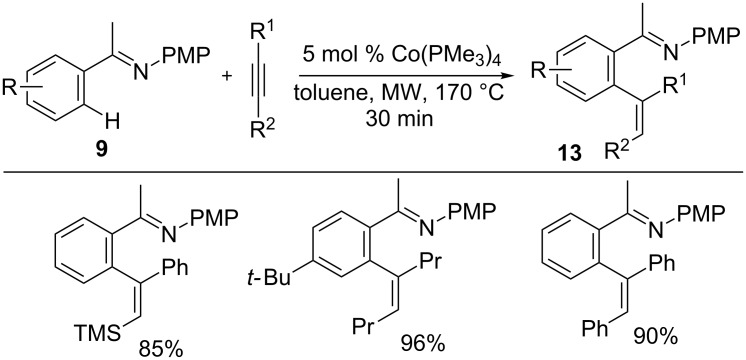
Co-catalyzed *anti*-selective C–H addition to alkynes.

In 2015, Petit’s group developed a hydroarylation reaction of internal alkynes with imines using a low-valent cobalt catalyst without reductant or additives (Scheme 10) [52]. The reaction afforded alkenes **13** in excellent yields with *anti*-selectivity. The alkene product originally should be *syn*-**13**, but it rearranged to *anti*-**13** at the high reaction temperature likely catalyzed by the cobalt complex. Moreover, the found intermolecular KIE of 1.4 and density functional theory (DFT) calculations strongly suggest that the reaction proceeds through concerted hydrogen transfer by an oxidative pathway, reductive elimination, and subsequent isomerization.

#### 1.2 Co(III)-catalyzed hydroarylation of alkynes

**Scheme 11:**
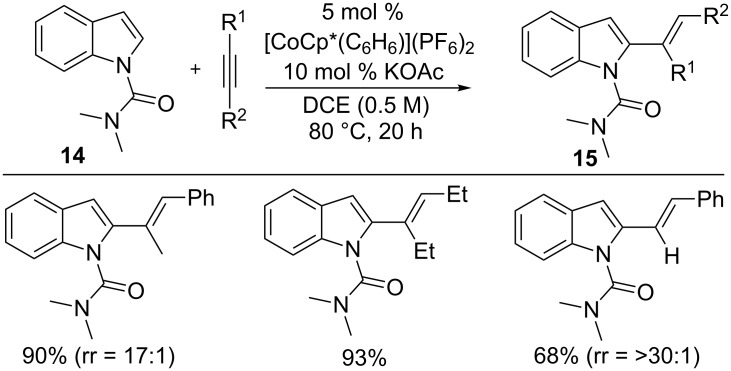
Co(III)-catalyzed hydroarylation of alkynes with indoles.

In 2013, Kanai/Matsunaga and co-workers developed an air-stable Co(III)Cp* catalyst as an economical alternative to Cp*Rh(III) for C–H functionalization [37]. The Co(III) catalyst was applied for the hydroarylation of alkynes with indoles **14** to form alkenes **15** with linear selectivity (Scheme 11) [53]. The reaction features a broad substrate scope including a variety of indoles, terminal and internal alkynes, mild reaction conditions and inexpensive catalyst. The reaction proceeds through an amide-assisted C–H metalation followed by alkyne insertion and protonation [54]. The reaction also acknowledged that the Co(III) species possess unique nucleophilic reactivity compared with the Rh(III) species for the annulation reaction. The hydroarylation reaction further extended to pyrroles for selective monoalkenylation using [Cp*Co(CH_3_CN)_3_](SbF_6_)_2_ as the catalyst [55].

**Scheme 12:**
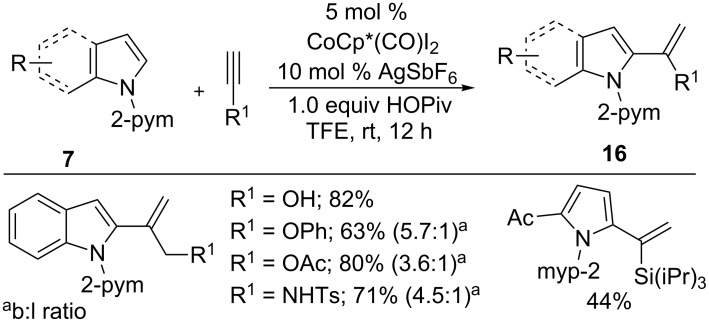
Co(III)-catalyzed branch-selective hydroarylation of alkynes.

In contrast, branched-selective hydroarylation of terminal alkynes was achieved by Li et al. The addition of arenes **7** to propargyl alcohols, protected propargyl amines, and silyl alkynes in the presence of CoCp*(CO)I_2_ catalyst gave uncommon branched-selective products **16** in good yields with reasonable selectivity (Scheme 12) [56]. DFT calculations indicated that the regioselectivity of silyl alkynes was controlled by its steric effect in the protonolysis step, whereas the electronic nature of propargyl alcohols and amines played a key role in the selectivity control during the insertion. Moreover, the control experiments and DFT calculations show that HOPiv played a crucial role in both the C−H activation and the protonolysis step.

**Scheme 13:**
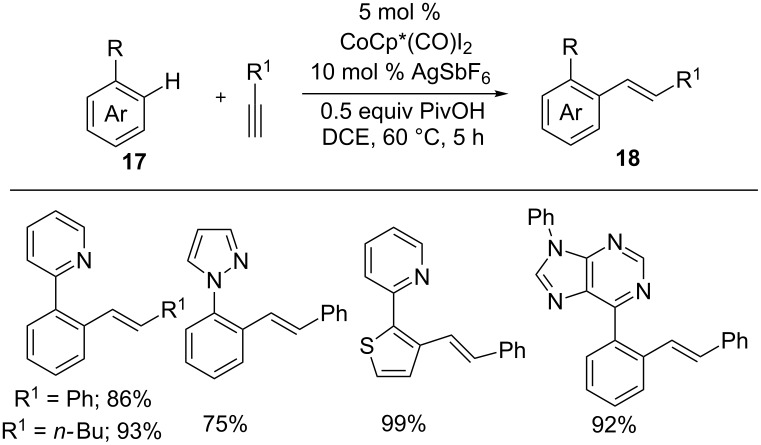
Co(III)-catalyzed hydroarylation of terminal alkynes with arenes.

In 2016, Yu and co-workers developed a hydroarylation of alkynes with different arenes including phenylpyridines, pyrazole, and 6-arylpurines **17** using 5 mol % Cp*Co(CO)I_2_, 10 mol % AgSbF_6_, and 0.5 equiv PivOH in DCE (Scheme 13) [57]. The reaction proceeded efficiently with various alkynes to give alkenes **18**, however, the reaction was limited to terminal alkynes. Additionally, they applied this methodology to design a mitochondria-targeted imaging dye from electron-withdrawing formyl-substituted indoles and alkynes.

**Scheme 14:**
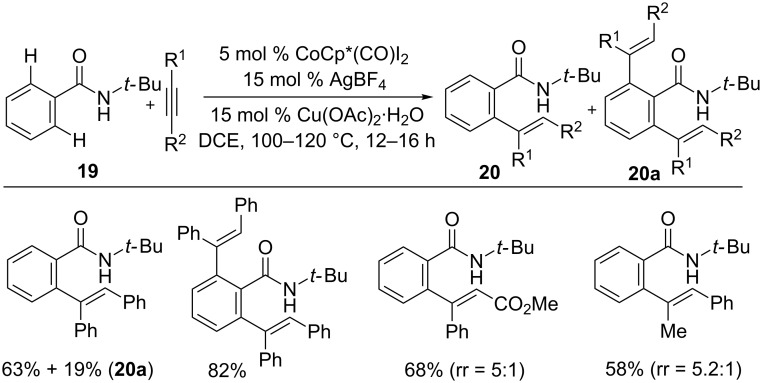
Co(III)-catalyzed hydroarylation of alkynes with amides.

Later, Maji’s group reported an *N*-*tert*-butyl amide-directed mono- and di-alkenylation reactions using a cobalt catalyst (Scheme 14) [58]. The reaction was also applied for the preparation of π-conjugated alkenes by four-fold C–H activation, which was found to be fluorescence active. The KIE studies provided *k*_H_/*k*_D_ of 2.8 and 2.6 through intermolecular and intramolecular experiments, respectively. These results strongly suggest that the C–H activation may be involved in the rate-limiting step. It is noteworthy that the reaction is limited to internal alkynes.

**Scheme 15:**
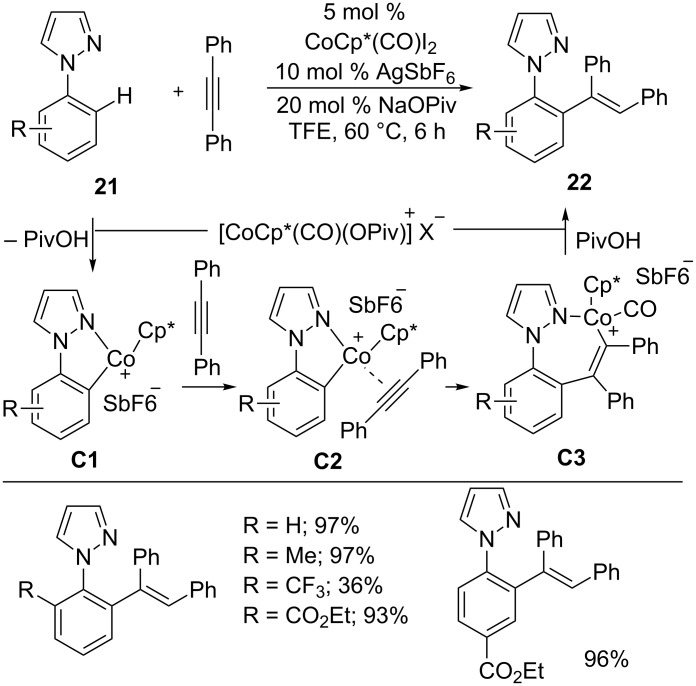
Co(III)-catalyzed C–H alkenylation of arenes.

Recently, Sundararaju and co-workers developed a Co-catalyzed hydroarylation reaction of alkynes with phenylpyrazoles **21** (Scheme 15) [59]. The reaction exhibited tolerance toward a variety of functionalities on arenes as well as alkynes. The isolated cationic cobalt intermediate (**C3**) and the mechanistic studies strongly indicate that the reaction proceeds through C–H activation via concerted metallation-deprotonation (CMD) to form cobaltacycle **C1.** Subsequently, alkyne coordination (**C2**) and migratory insertion of alkyne provide alkenyl intermediate **C3**. Finally, protonolysis gives the desired alkene product **22** and regenerates the active cobalt(III) catalyst for the next cycle.

### 2. Hydroarylation of alkenes

#### 2.1 Low-valent cobalt-catalyzed hydroarylation of alkenes

**Scheme 16:**
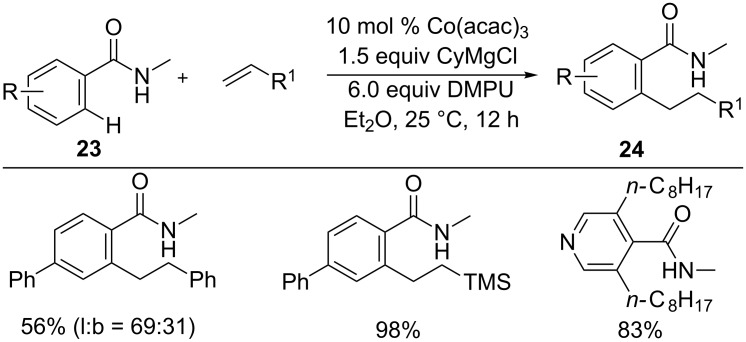
Co-catalyzed alkylation of substituted benzamides with alkenes.

As alkynes efficiently participated in low-valent cobalt-catalyzed hydroarylations (section 1.1), Nakamura and co-workers developed an analogous hydroarylation reaction of alkenes [39]. Thus, the reaction of amides **23** with alkenes in the presence of 10 mol % Co(acac)_3_, 1.5 equiv CyMgCl, and 6.0 equiv DMPU provided C–H alkylated products **24** in good yields with high linear selectivity (Scheme 16).

**Scheme 17:**
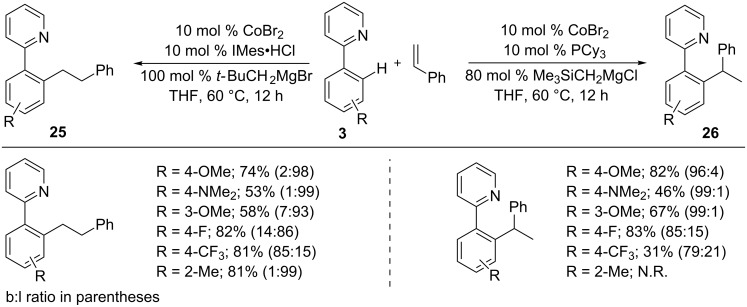
Co-catalyzed switchable hydroarylation of styrenes with 2-aryl pyridines.

Later, Yoshikai’s group reported a ligand-controlled hydroarylation of styrenes with 2-phenylpyridines **3** in the presence of CoBr_2_-IMes and CoBr_2_-PCy_3_ catalysts to synthesize linear and branched selective products **25** and **26**, respectively, in good yields with high selectivity control (Scheme 17) [60]. It is noteworthy that the electronic nature of the substrates also controlled the addition selectivity; electron-withdrawing group (CF_3_ and F) substituted arenes **3** favored branched addition product **26** in both catalytic systems.

**Scheme 18:**
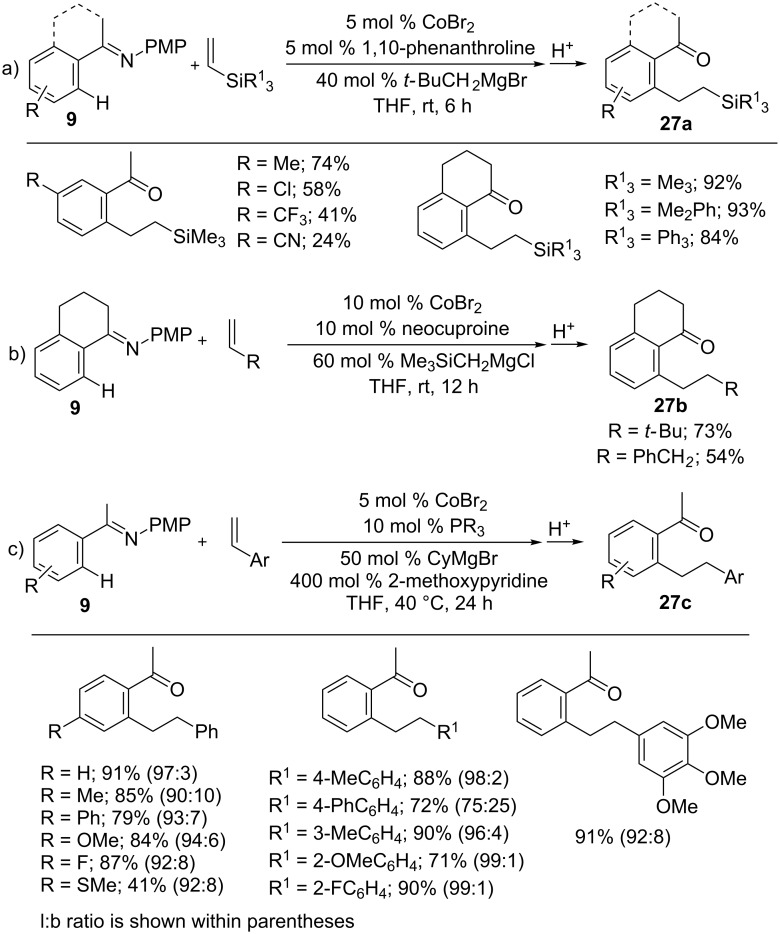
Co-catalyzed linear-selective hydroarylation of alkenes with imines.

Similarly, imines **9** were employed for the hydroarylation reaction to form linear or branched addition products by tuning ligands and additives. Thus, the reaction of imines **9** with vinylsilanes as well as alkyl alkenes gave linear addition products **27a,b** under mild reaction conditions (Scheme 18a,b) [61]. The addition of indoles to vinylsilanes also succeeded in a similar manner [62]. When styrenes were employed for the hydroarylation with imines **9**, the reaction required 4.0 equiv of 2-methoxypyridine as the additive in the presence of CoBr_2,_ [bis(2,4-dimethoxyphenyl)(phenyl)phosphine] and CyMgBr to afford linear-selective products **27c** (Scheme 18c) [63].

**Scheme 19:**
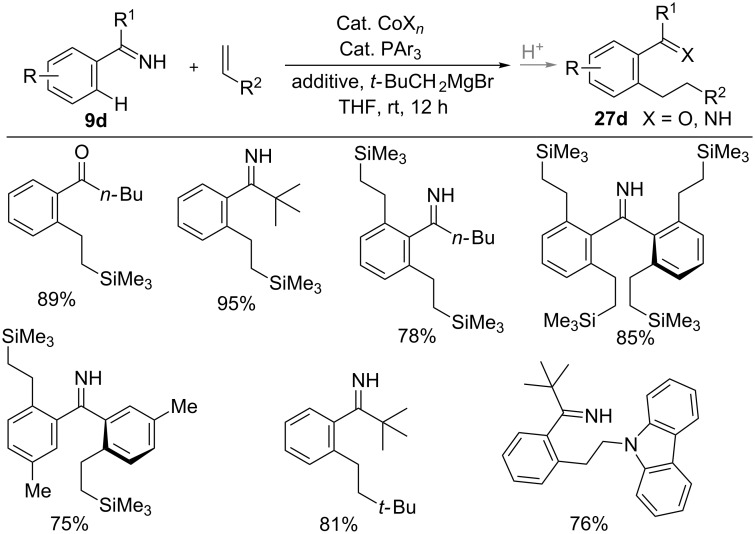
Co-catalyzed linearly-selective hydroarylation of alkenes with N–H imines.

Recently, N–H imines **9d** has also become an efficient directing group for the hydroarylation reaction. The addition of N–H imines **9d** to vinylsilanes or alkyl alkenes was achieved using a low-valent cobalt catalyst to form alkylated products **27d** (Scheme 19) [64]. The N–H imines overcame substrate scope limitation from previous reports with *N*-aryl and *N*-alkylimines. Moreover, multiple C–H alkylation was also succeeded with benzophenone imines in high yields with linear selectivity under the mild reaction conditions.

**Scheme 20:**
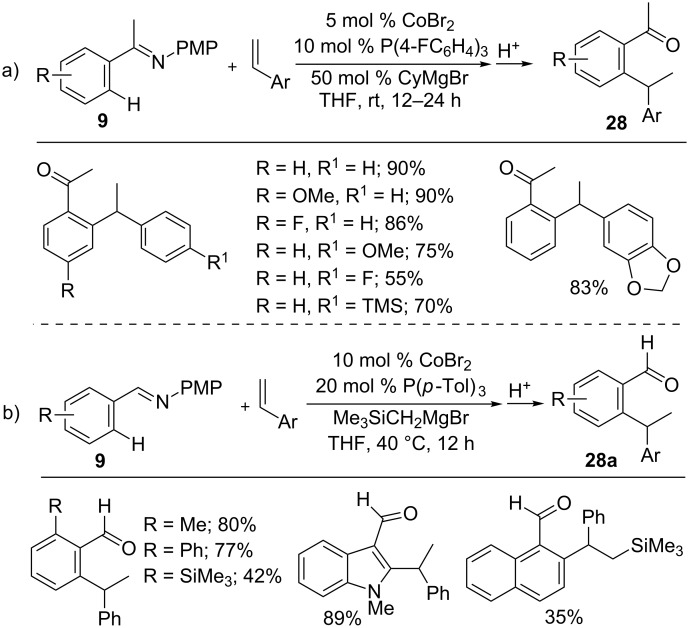
Co-catalyzed branched-selective hydroarylation of alkenes with imines.

In contrast, the addition of ketimines **9** to styrenes gave branched selective alkylation products **28** in the presence of the catalyst system CoBr_2_/P(4-FC_6_H_4_)_3_ and CyMgBr (Scheme 20a) [65]. In a similar manner, aldimines **9** also reacted with styrenes to give branched-selective alkyls **28a** under modified reaction conditions (Scheme 20b) [66]. Moreover, the Co-catalyzed hydroarylation of styrene with ketimine or aldimine proceeded without Grignard reagent using Mg metal as the reductant [67]. Recently, N–H imines **9d** were also employed for the hydroarylation reaction with styrenes, giving a branched-selective hydroarylation product in good yields [68].

**Scheme 21:**
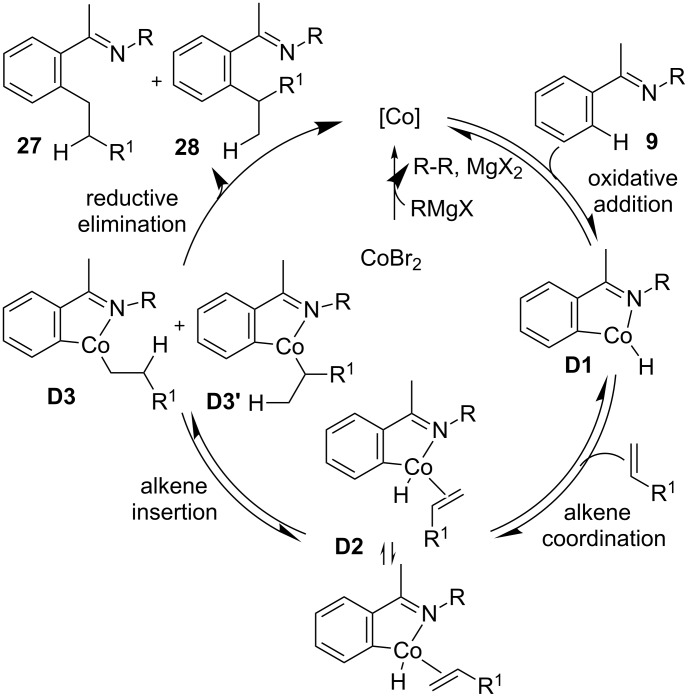
Mechanism of Co-catalyzed hydroarylation of alkenes.

Based on the mechanistic studies and DFT calculations [69], a plausible mechanism for the cobalt-catalyzed ligand-controlled hydroarylation of alkenes was proposed in Scheme 21. The reaction begins with the generation of low-valent cobalt from CoBr_2,_ ligand, and Grignard reagent. Then, imine **9** assisted an *ortho* C–H metallation by oxidative addition and provides Co–H intermediate **D1**. Coordination of alkene with **D1** and insertion of the alkene to Co–H gives intermediate **D3** or **D3’**, which is converted into product **27** or **28** and low-valent cobalt by reductive elimination.

**Scheme 22:**
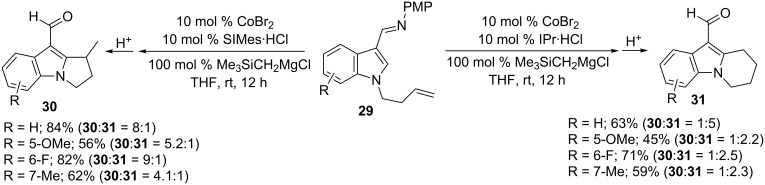
Co-catalyzed intramolecular hydroarylation of indoles.

Indoles also efficiently participated in intra- and intermolecular hydroarylation reactions to access useful organic skeletons [70–73]. Thus, the reaction of indole bearing an aldimine and homoallyl group **29** in the presence of CoBr_2_, SIMes·HCl and Me_3_SiCH_2_MgCl gave dihydropyrroloindoles **30**, whereas the IPr·HCl ligand provided tetrahydropyridoindoles **31** in reasonable regioselectivity (Scheme 22) [70]. Remarkably, the regioselectivity of the reaction is not only controlled by the steric effect of NHC ligand, but also depends on the olefin tether in **29**. Recently, Petit and co-workers also developed an intra- and intermolecular hydroarylation of indoles using Co(PMe_3_)_4_ as the catalyst under Grignard-free conditions [72].

**Scheme 23:**
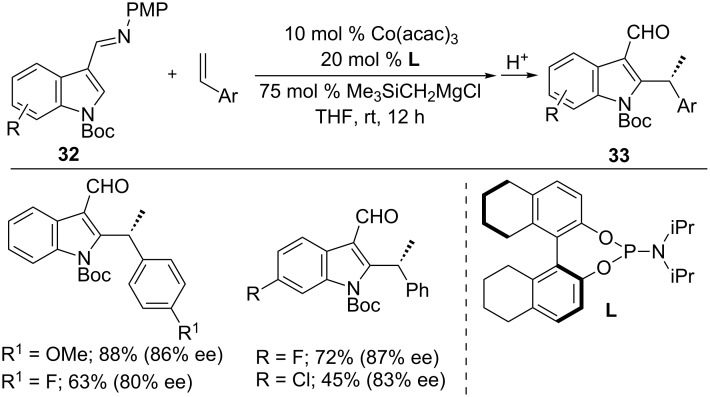
Co-catalyzed asymmetric hydroarylation of alkenes with indoles.

In 2015, an asymmetric hydroarylation of styrenes with indoles was reported by Yoshikai and co-workers (Scheme 23) [73]. Among varies *N*-protected indoles, *N*-Boc indoles **32** in the presence of Co(acac)_3_ and phosphoramidite ligand **L** afforded alkylated products **33** in high yields and enantioselectivity. A wide range of indoles and styrenes were well tolerated to form the corresponding alkyl products **33** in a branched selective manner.

**Scheme 24:**
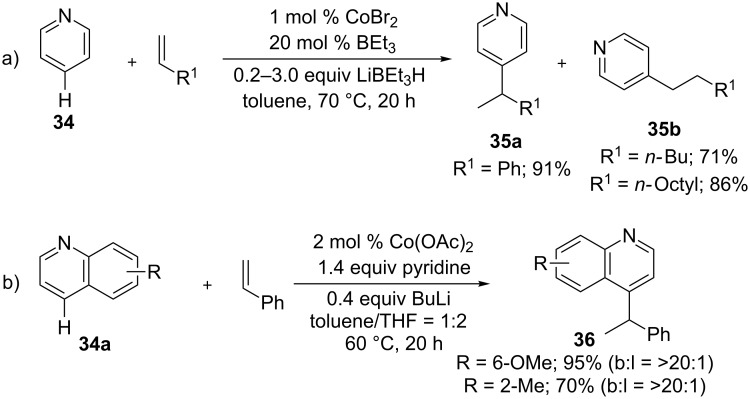
Co-catalyzed hydroarylation of alkenes with heteroarenes.

In addition to the directing group strategies, remote C4-selective alkylation of pyridines [74] was also feasible with alkenes as Kanai et al. reported (Scheme 24a) [75]. The addition of pyridine (**34**) to alkenes in the presence of 1 mol % CoBr_2_, 20 mol % BEt_3_, and LiBEt_3_H as a hydride source provided branched-selective products **35a** with styrenes, while alkylalkenes resulted in linear-selective products **35b**. Similarly, quinolines **34a** also underwent hydroarylation reaction with styrene to give C4-selective alkylated products **36** in good regioselectivity (Scheme 24b) [76].

#### 2.2 Co(III)-catalyzed hydroarylation of alkenes

**Scheme 25:**
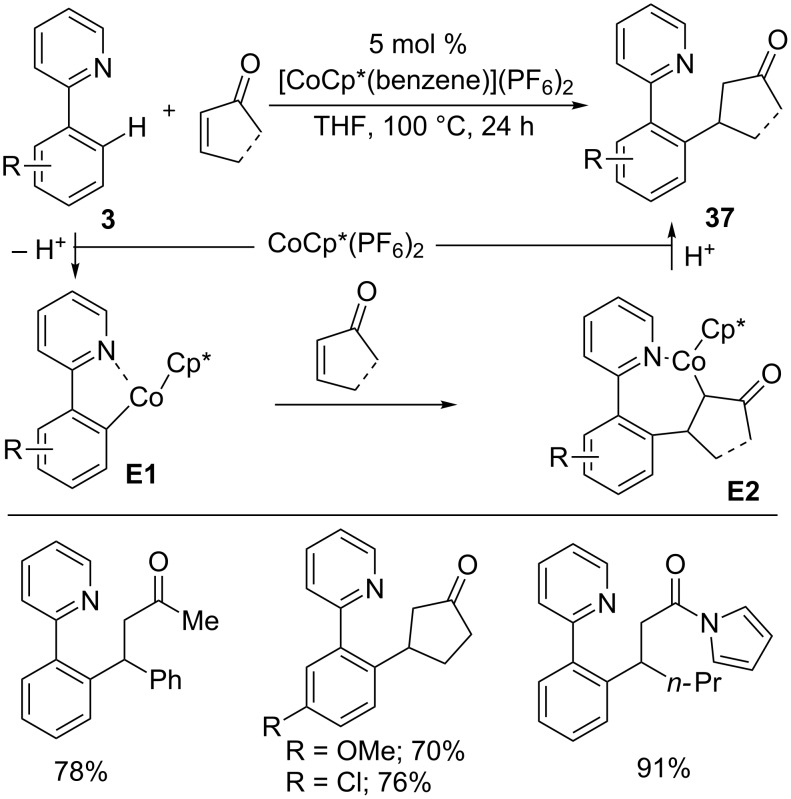
Co(III)-catalyzed hydroarylation of activated alkenes with 2-phenyl pyridines.

In 2013, Kanai/Matsunaga and co-workers reported a hydroarylation reaction of activated alkenes with phenyl pyridines **3** in the presence of the air-stable [Co(III)Cp*(benzene)](PF_6_)_2_ catalyst, giving hydroarylation products **37** in good yields (Scheme 25) [37]. The reaction proceeds through directed *ortho* C–H metallation to form five-membered cobaltacycle **E1** and alkene insertion to give complex **E2**. Protonation then provides product **37** and an active Co(III) catalyst for the next cycle.

**Scheme 26:**
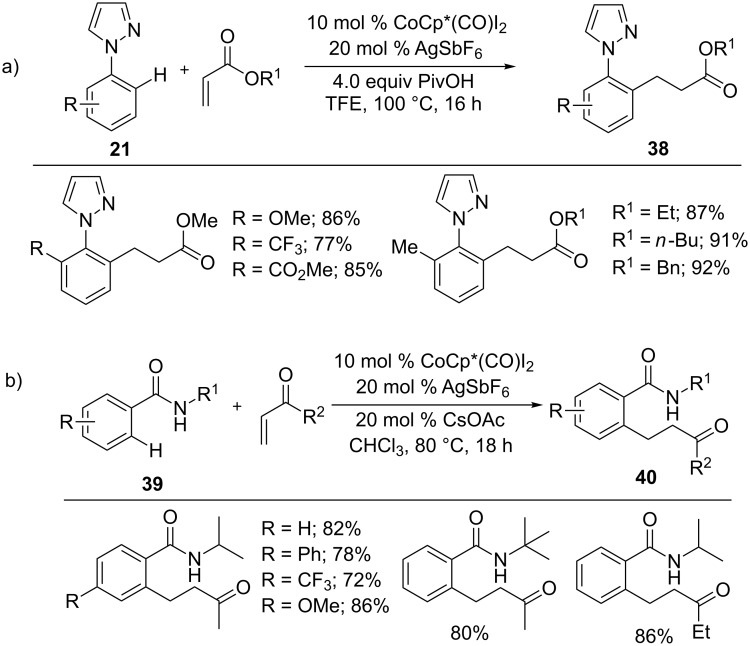
Co(III)-catalyzed C–H alkylation of arenes.

Terminal alkenes were also applied in hydroarylation reaction as Sundararaju and co-workers demonstrated (Scheme 26a) [77]. Likewise, Whiteoak’s group reported a cobalt-catalyzed hydroarylation of terminal alkenes with amides **39** [78]. The reaction of vinyl ketones with amides provided alkylated product **40**, whereas acrolein resulted in biologically useful azepinones in good yields (Scheme 26b).

**Scheme 27:**
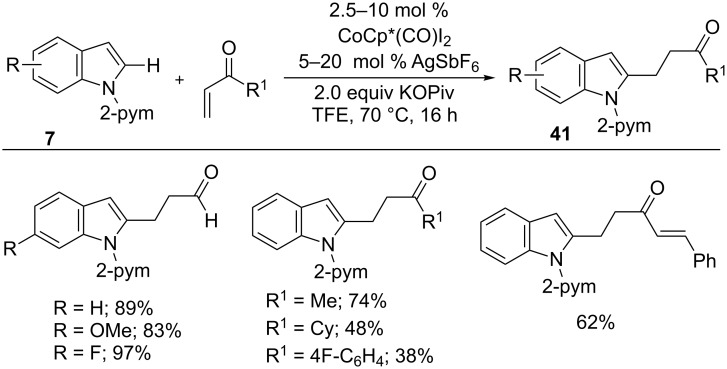
Co(III)-catalyzed C2-alkylation of indoles.

*N*-Pyrimidylindole is a highly reactive arene in cobalt-catalyzed C–H functionalizations. In this context, Li and co-workers developed an addition reaction of indoles **7** with activated alkenes using a cobalt(III) catalyst (Scheme 27) [79]. The reaction tolerated a wide range of alkenes including vinyl aldehyde, ketones, and divinyl ketones and a variety of arenes to give C2-alkylation products **41** in good yields.

**Scheme 28:**
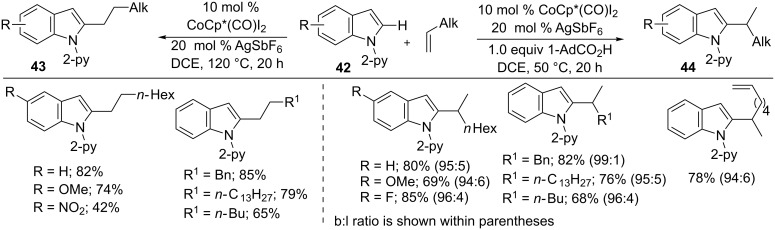
Co(III)-catalyzed switchable hydroarylation of alkyl alkenes with indoles.

Similarly, alkylalkenes were subjected to hydroarylation with indoles by Ackermann et al. [80]. The addition of indoles **42** into alkylalkenes in the presence of 10 mol % CoCp*(CO)I_2_, 20 mol % AgSbF_6_ at 120 °C gave linear-selective products **43**, whereas additional 1.0 equiv 1-AdCO_2_H at 50 °C resulted in predominantly branched-selective products **44** (Scheme 28). The reaction features switchable regioselectivity, a broad scope, and an inexpensive catalyst. Moreover, DFT calculations and mechanistic studies revealed that the switchable regioselectivity was driven by a change in mechanism from linear ligand-to-ligand hydrogen transfer to branched base-assisted internal electrophilic-type substitution.

**Scheme 29:**
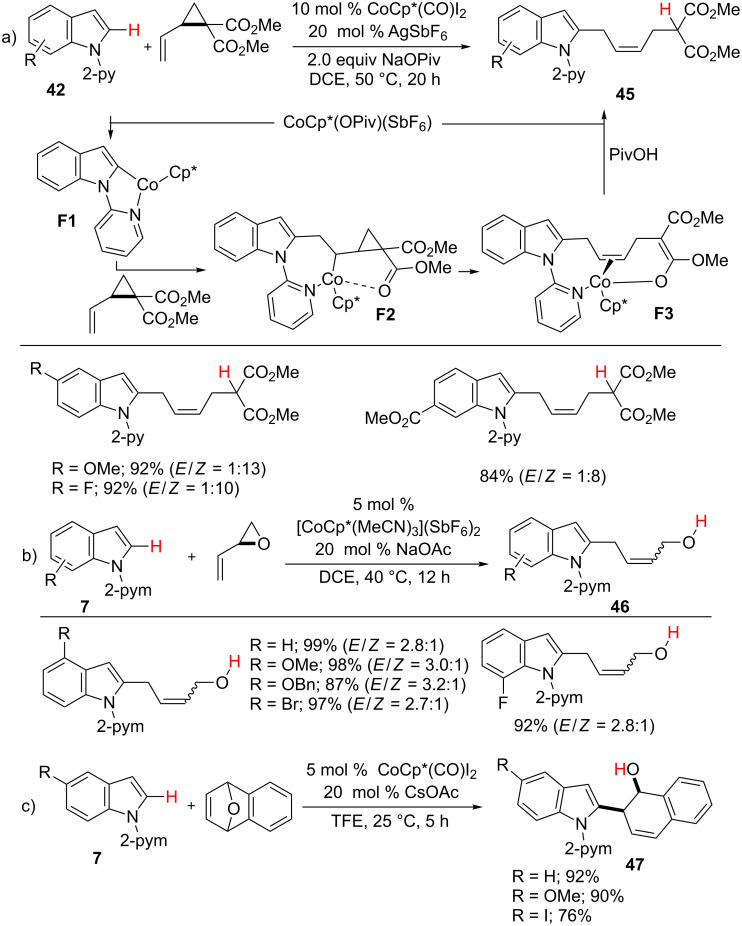
Co(III)-catalyzed C2-allylation of indoles.

In addition to activated and unactivated alkylalkenes, vinylcyclopropanes also underwent hydroarylation reactions with indoles **42** via C–H/C–C activation (Scheme 29a) [81]. The reaction afforded allylated products **45** in high stereoselectivities. DFT calculations and mechanistic studies strongly indicate that the reaction proceeds through the generation of complex **F1** by pyridine-directed C–H activation. Subsequently, alkene insertion with **F1** and C–C activation of **F2** give intermediate **F3**. Finally, protonolysis provides the desired product **45** and an active Co(III)Cp*. Due to the low activation energy, the thermodynamically less stable *Z* diastereomer is preferred in the reaction, which is in contrast to the rhodium(III)-catalyzed reaction [82]. Later, Li and co-workers developed a cobalt-catalyzed hydroarylation of 2-vinyloxiranes with indoles **7** to form C2-allylated products **46**, albeit in low stereoselectivity (Scheme 29b) [83]. Similarly, Cheng et al. reported a hydroarylation reaction of arenes **7** with a bicyclic alkene to form ring-opening products **47** via C–H/C–O activation (Scheme 29c) [84]. The product **47** was further converted into the C–H naphthylation product in the presence of an acid.

**Scheme 30:**
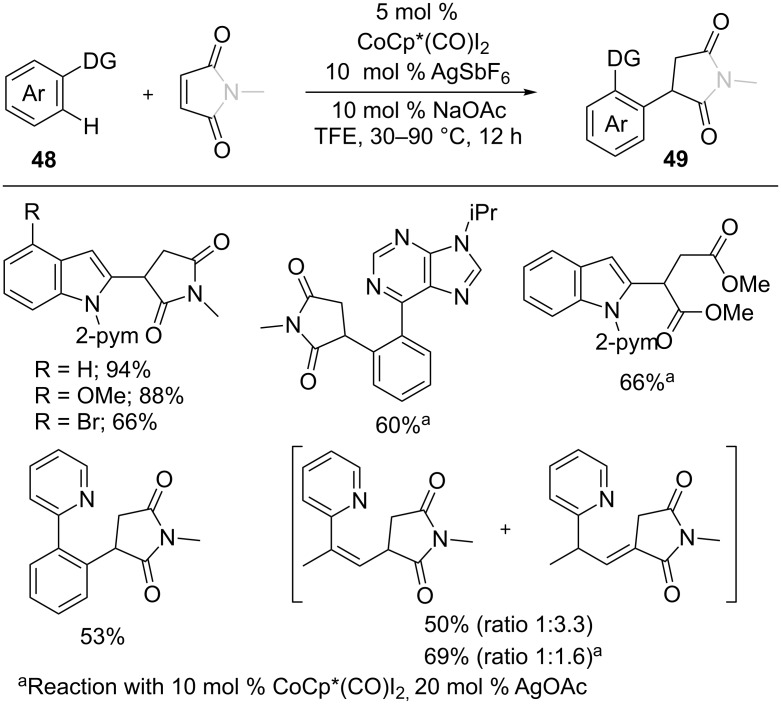
Co(III)-catalyzed *ortho* C–H alkylation of arenes with maleimides.

Maleimides turned out to be an efficient coupling partner in C–H functionalization to synthesize biologically useful succinimides in highly atom-economical manner. Thus, Li et al. developed a cobalt-catalyzed hydroarylation of maleimides and maleate esters with arenes **48** (Scheme 30) [85]. A variety of arenes including indoles, 2-arylpyridines, 6-arylpurine, and vinyl pyridines were employed to give alkylated products **49** in good to moderate yields. As well, Prabhu and co-workers also reported a Co-catalyzed hydroarylation reaction of maleimides with indoles [86].

**Scheme 31:**
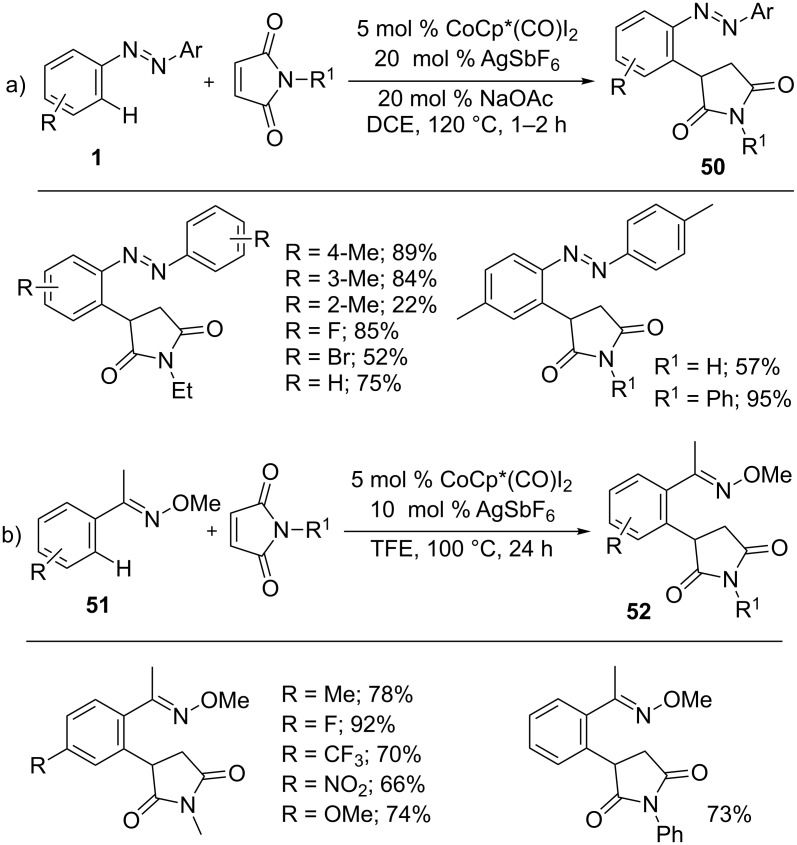
Co(III)-catalyzed hydroarylation of maleimides with arenes.

The hydroarylation of maleimides was further demonstrated with different arenes (Scheme 31). Thus, azobenzenes **1** were subjected to alkylation reaction with maleimides to form a variety of succinimides **50** in good yields (Scheme 31a) [87]. Likewise, oximes **51** [88] were also efficient participated in the hydroarylation of maleimides (Scheme 31b).

### 3. Hydroarylation of allenes

**Scheme 32:**
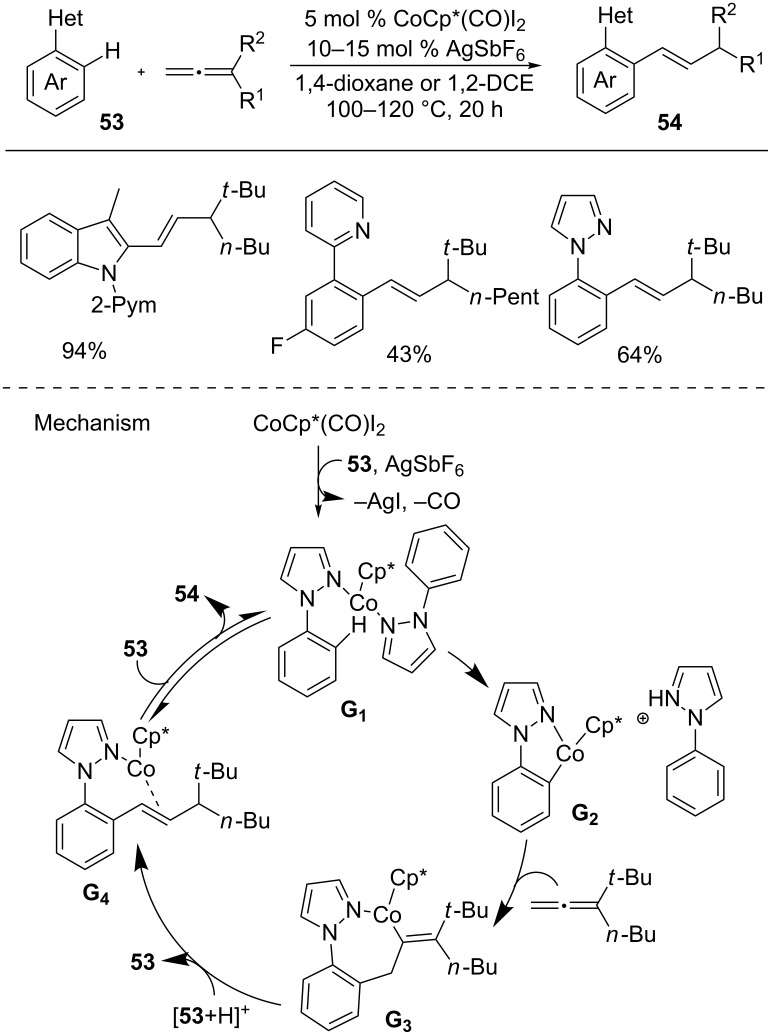
Co(III)-catalyzed hydroarylation of allenes with arenes.

An allene is an exceptional functional group in organic synthesis due to its cumulative double bonds [89–90]. In this context, Ackermann and co-workers developed a coupling reaction of arenes **53** with allenes to give hydroarylation products **54** (Scheme 32) [91]. The merits of the reaction are broad scope including a variety of arenes and allenes, and high atom economy. Based on the mechanistic studies and DFT calculations, a plausible mechanism was proposed. The reaction starts with the generation of the active cobalt(III) species **G1** from [Cp*CoI_2_(CO)], AgSbF_6_, and arene **53**. Subsequently, C–H metallation of **G1** by ligand-to-ligand hydrogen transfer provides cobaltacycle **G2** and an allene insertion gives intermediate **G3**. Protonation and isomerization generate cobalt complex **G4**, which is converted into alkenated product **54** by ligand exchange.

### 4. Hydroarylation of enynes

**Scheme 33:**
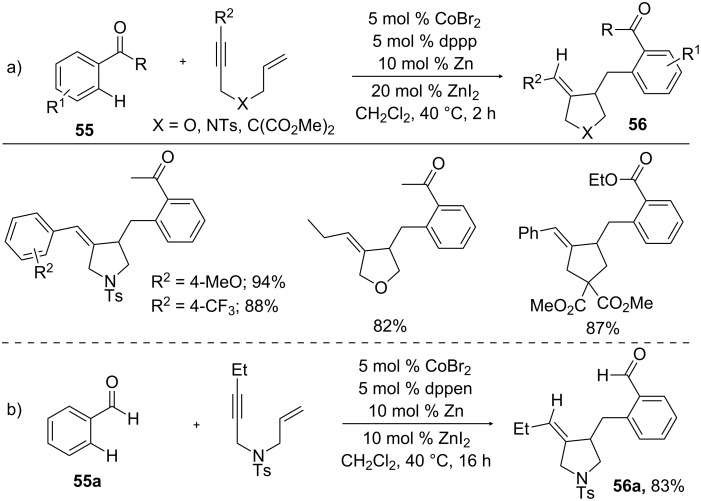
Co-catalyzed hydroarylative cyclization of enynes with carbonyl compounds.

Catalytic cyclization of 1,*n*-enynes has become as an attractive tool for the preparation of cyclic adducts with a variety of functionalities in a one-pot process [92]. As the cyclization of 1,*n*-enynes with organometallics is well-known, the addition of an inert C–H bond to 1,*n*-enynes further enhances the economy of cyclization process. Thus, Cheng and co-workers reported a cobalt-catalyzed hydroarylative cyclization of 1,*n*-enynes with carbonyl compounds **55** to form a wide range of functionalized dihydrofurans and pyrrolidines **56** in good yields (Scheme 33a) [93]. By tuning the diphosphine ligands, the reaction was extended to aromatic aldehyde **55a**, where slightly electron-deficient ethylene diphosphine ligand delivered hydroarylation product **56a**, but a mild electron-rich ligand resulted in hydroacylation product (Scheme 33b) [94].

**Scheme 34:**
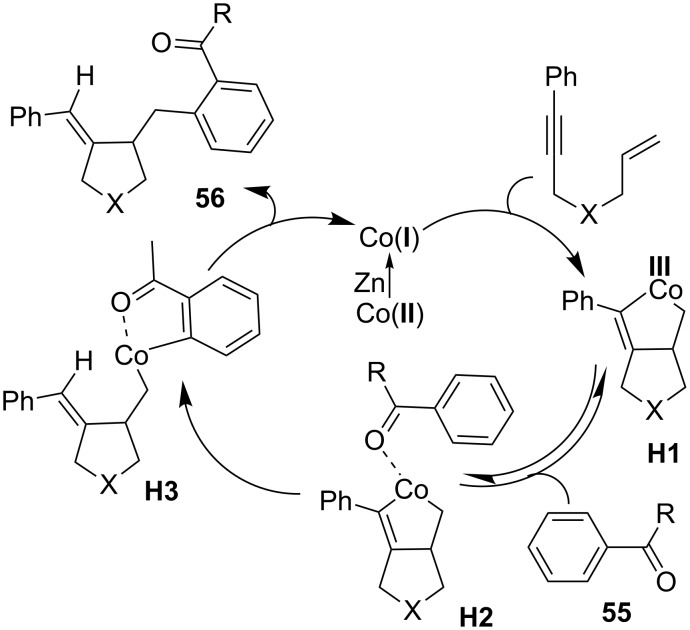
Mechanism for the Co-catalyzed hydroarylative cyclization of enynes with carbonyl compounds.

A plausible mechanism for the hydroarylative cyclization of enynes was shown in Scheme 34. The reaction begins with the reduction of Co(II) to Co(I) by Zn dust. The enyne compound underwent oxidative addition with Co(I) to form bicyclic cobaltacycle **H1**. After the reversible coordination of arene **55** with **H1** to generate intermediate **H2**, *ortho* C–H cobaltation provides complex **H3**, which changed into product **56** and Co(I) catalyst by reductive elimination of **H3**.

### 5. Hydroarylation of C=X (X = N, O) bonds

#### 5.1 Low-valent cobalt-catalyzed hydroarylation of C=X bonds

**Scheme 35:**
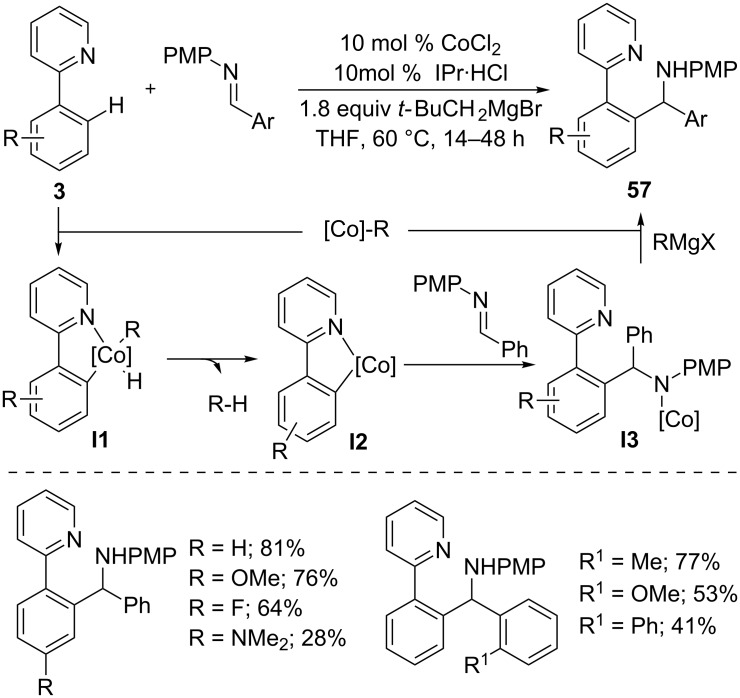
Co-catalyzed addition of 2-arylpyridines to aromatic aldimines.

Transition-metal-catalyzed addition of C–H bond to polar π bonds such as imines, isocyanates and carbonyls is one of the efficient methods to incorporate heteroatoms in organic molecules [45]. Yoshikai et al. developed an addition reaction of arylpyridines **3** to imines using low-valent cobalt catalyst generated from CoCl_2_, IPr**·**HCl, and *t-*BuMgBr (Scheme 35) [95]. The reaction tolerated a wide range of arylpyridines and aldimines, giving diarylmethylamines **57** in good yields. The reaction possibly proceeds through the formation of neopentylcobalt ([Co]-R), which undergoes oxidative addition with arylpyridine to generate **I1**. Then, elimination of neopentane (R–H) by reductive elimination gives cobaltacycle **I2**. Nucleophilic addition of **I2** to imines, followed by transmetallation with a Grignard reagent and protonation provide the desired hydroarylation product **57**.

**Scheme 36:**
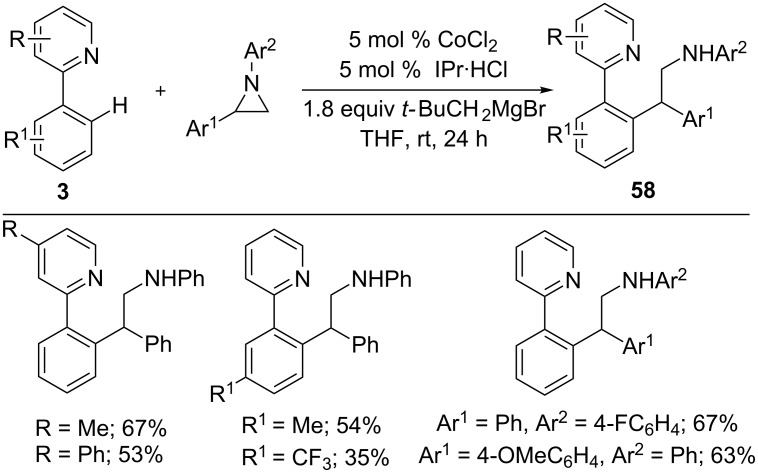
Co-catalyzed addition of 2-arylpyridines to aziridines.

In addition to imines, aziridines were also amenable to the cobalt-catalyzed hydroarylation reaction (Scheme 36) [96]. Treatment of 2-phenylpyridines **3** with varies aryl-substituted aziridines gave 1,1-diarylethane derivatives **58** in a highly regioselective manner. It is noteworthy that nucleophilic addition took place selectively at the more hindered C-2 position of aziridines, which results in high regioselectivity.

#### 5.2 Cobalt(III)-catalyzed hydroarylation of C=X bonds

**Scheme 37:**
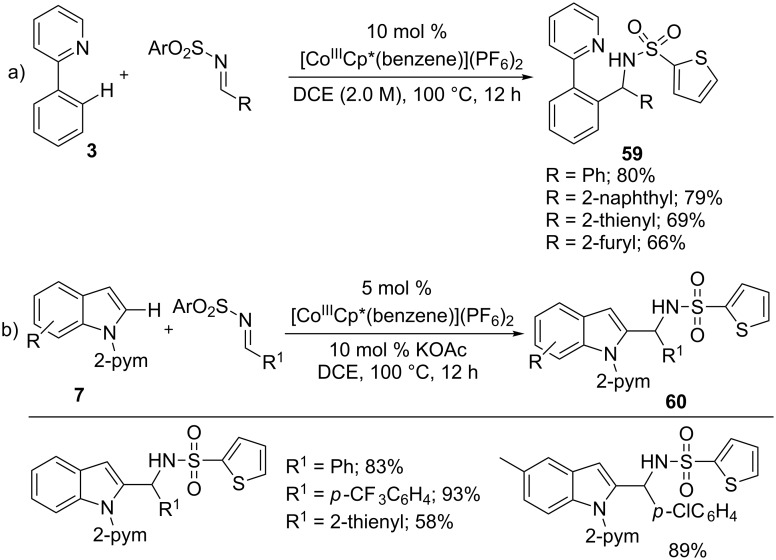
Co(III)-catalyzed hydroarylation of imines with arenes.

As the Co(III)Cp* catalyst acts an efficient air-stable catalyst for hydroarylation of the C=C bond, it would also provide a user-friendly method for C=X bonds. Thus, imines were employed for hydroarylation reaction with 2-phenylpyridines **3** in the presence of [CoCp*(benzene)](PF_6_)_2_ catalyst by Kanai/Matsunaga and co-workers (Scheme 37a) [37]. The reaction afforded diarylmethylamines **59** in good to moderate yields, however, the imines used was limited to aldimines. Similarly, indoles **7** also efficiently underwent the hydroarylation reaction with various substituted aldimines to provide C-2 alkylated indoles **60** (Scheme 37b) [97].

**Scheme 38:**
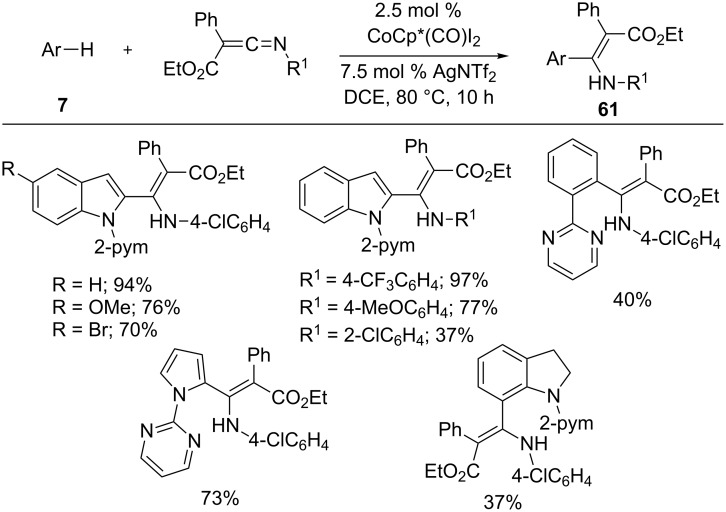
Co(III)-catalyzed addition of arenes to ketenimines.

In 2016, Wang’s group demonstrated that ketenimines participated in hydroarylation reaction with arenes **7** in the presence of CoCp*(CO)I_2_ and AgNTf_2_ (Scheme 38) [98]. The reaction tolerated various pyrimidine containing arenes, such as indole, phenyl, and pyrrole with different ketenimines to form enaminylation products **61**. Subsequently, the hydroarylation products **61** were further converted into bioactive pyrrolo[1,2-*a*]indoles by sodium ethoxide-promoted cyclization.

**Scheme 39:**
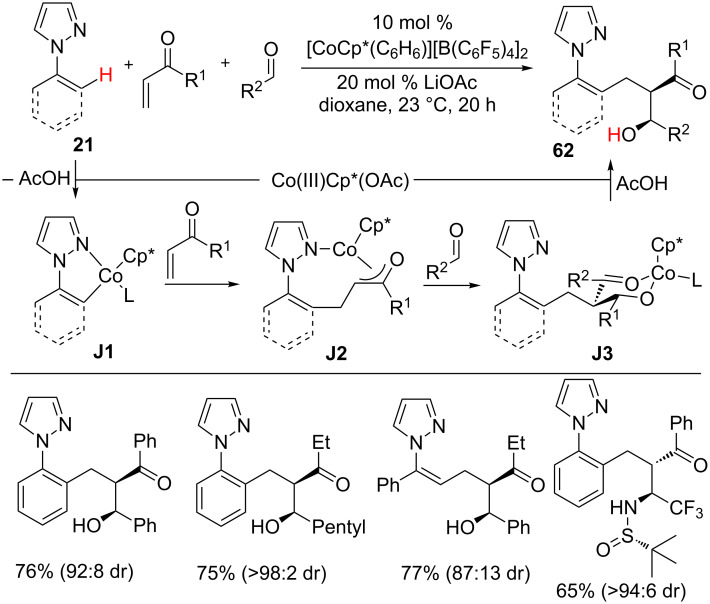
Co(III)-catalyzed three-component coupling.

**Scheme 40:**
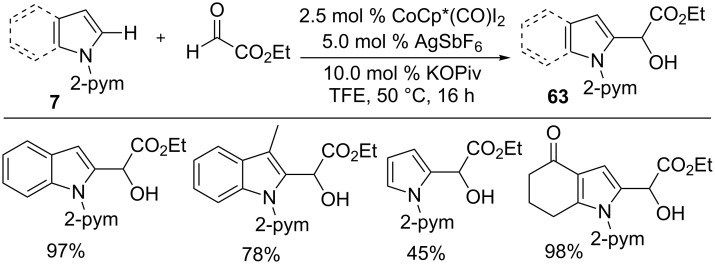
Co(III)-catalyzed hydroarylation of aldehydes.

The three-component coupling also becomes viable through a Co(III)-catalyzed hydroarylation strategy as Ellman and co-workers demonstrated (Scheme 39) [99]. The reaction of arene **21** with vinyl ketones and aldehydes in the presence of [CoCp*(C_6_H_6_)][B(C_6_F_5_)_4_]_2_ and LiOAc gave alcohol products **62** with high diastereoselectivity at ambient reaction conditions. The unique nature of cobalt was well presented by its superior reactivity and diastereoselectivity and it outmatched the rhodium catalyst. Different pyrazole derivatives with a wide range of aldehydes or imines were participated well in the reaction, affording the addition products **62** in good yields. The catalytic cycle starts with the coordination of active cobalt catalyst with arene **21** followed by C–H activation to give five-membered cobaltacycle **J1**. Insertion of the alkene with **J1** forms cobalt enolate **J2**, which converts into **J3** by the addition of aldehyde via a chair transition state in a diastereoselective manner. Finally, protonolysis affords product **62** and regenerates the active Co(III)Cp* catalyst for the next cycle. Later, Li et al. reported a Co(III)-catalyzed hydroarylation of glyoxylate with pyrimidine containing indoles and pyrroles **7** to provide products **63** with high productivity (Scheme 40) [79].

**Scheme 41:**
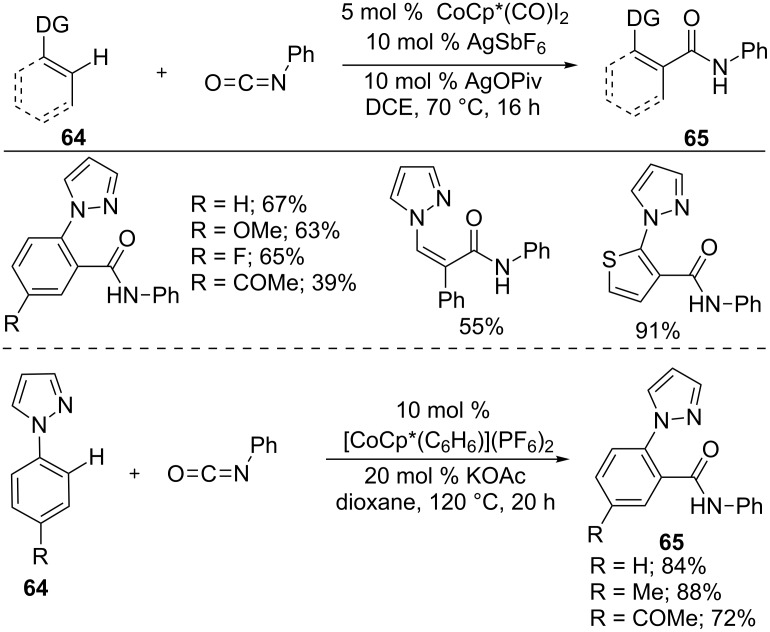
Co(III)-catalyzed addition of arenes to isocyanates.

Similar to the imine, isocyanate is also an efficient electrophile for hydroarylation of C=N bond. It provides a high atom- and step-economical method for the preparation of aromatic, vinyl, and heterocyclic amides. Thus, Ackermann and co-workers developed an inexpensive cobalt-catalyzed hydroarylation of isocyanates with (hetero)arenes **64** (Scheme 41) [100]. The reaction can tolerate a broad range of arenes and isocyanates, providing amide products **65** with notable site selectivity. The found inter- and intramolecular KIEs (*k*_H_/*k*_D_) of 1.4 shows that the C−H activation may not be the rate the determining step. Moreover, competition experiments indicate that the reaction favors electron-rich arenes **64** and electron-deficient isocyanates. Ellman’s group also reported a similar C–H amidation reaction of aryl pyrazoles **64** with isocyanates in the presence of [CoCp*(C_6_H_6_)](PF_6_)_2_ and KOAc [101].

## Conclusion

Hydroarylation is an emerging methodology in organic synthesis, because it is a highly atom-, step-, and redox-economical and simple reaction compared to traditional synthetic methods to construct new C−C bonds. Among the first-row transition metals, both low- and high-valent cobalt catalysts have played a substantial role in these hydroarylation reactions providing an economical alternative to the precious metal-catalyzed reactions and also showed distinct selectivity in some cases. Switchable regioselective hydroarylation of styrene using low-valent cobalt catalyst and Co(III)-catalyzed hydroarylation of alkylalkenes with indoles are remarkable examples in this manner. A wide range of C−H bonds has been successfully added to alkynes, alkenes, imines etc. to build alkyls, alkenes, alcohols, amides, and cyclic skeletons with excellent efficacy. Considering the importance of green processes for the ever-growing universe, economical and waste-free hydroarylation strategy will continue to draw great attention in the field of organic synthesis.
